# Renal Transplant Pathology: Demographic Features and Histopathological Analysis of the Causes of Graft Dysfunction

**DOI:** 10.1155/2020/7289701

**Published:** 2020-12-07

**Authors:** Shaarif Bashir, Mudassar Hussain, Azhar Ali Khan, Usman Hassan, Khawaja Sajid Mushtaq, Maryam Hameed, Usman Ayub Awan

**Affiliations:** ^1^Department of Pathology, Shaukat Khanum Memorial Cancer Hospital and Research Centre, Lahore 54000, Pakistan; ^2^Department of Nephrology, Shaikh Zayed Hospital, Lahore 54000, Pakistan

## Abstract

**Background:**

Renal transplant has emerged as a preferred treatment modality in cases of end-stage renal disease; however, a small percentage of cases suffer from graft dysfunction.

**Aim:**

To evaluate the renal transplant biopsies and analyze the various causes of graft dysfunction.

**Materials and Methods:**

163 renal transplant biopsies, reported between 2014 and 2019 and who fulfilled the inclusion criteria, were evaluated with respect to demographics, clinical, histological, and immunohistochemical features.

**Results:**

Of 163 patients, 26 (16%) were females and 137 (84%) were males with a mean age of 34 ± 7 years. 53 (32.5%) cases were of rejection (ABMR and TCMR), 1 (0.6%) was borderline, 15 were of IFTA, and rest of 94 cases (57.7%) belonged to the others category. SCr (serum creatinine) in cases of rejection was 3.85 ± 0.55 mg/dl. Causes of early graft dysfunction included active ABMR (7.1 ± 4.7 months), acute TCMR (5.5 months), and acute tubular necrosis (after 6 ± 2.2 months of transplant) while the causes of late rejection were CNIT and IFTA (34 ± 4.7 and 35 ± 7.8 months, respectively).

**Conclusion:**

Renal graft dysfunction still remains a concerning area for both clinicians and patients. Biopsy remains the gold standard for diagnosing the exact cause of graft dysfunction and in planning further management.

## 1. Introduction

Renal transplantation has emerged as a favored treatment modality for patients with end-stage renal disease [1]. However, various factors influence graft functioning and various causes can lead to graft dysfunction. Graft dysfunction is suspected when there is rising serum creatinine, proteinuria, active urinary sediment, and uncontrolled hypertension [2]. Most common causes include immunological injuries like ABMR/TCMR, recurrence or de novo glomerulonephritis, and thrombotic microangiopathy [1, 2]. Definite diagnosis of graft impairment requires histological examination of biopsies from the transplanted kidney [1].

There has been a 1.6 fold increase in the total number of renal transplantations in Asian population in the past decade with 994 transplants in 2005 and 1661 transplants in 2015 in Japan [1]. Another study in Iran reported a significant increase in the number of ESRD patients undergoing renal replacement therapy from 238 per million populations (pmp) in 2000 to 435.8 pmp in 2007 [3]. Despite developments of in-hospital services and patient care, there are still a significant number of graft dysfunction cases.

A uniform approach to interpret renal transplant pathologies was developed in 1991, by Prof. Kim Solez, as Banff classification of Allograft pathology [4]. Since then, a total of 15 meetings, reflected in multiple articles, have improved upon it [4, 5]. The latest meeting was held in 2019 at Pittsburgh, and the updated Banff classification (2019) is now being used all over world [5].

According to Banff classification, two major causes of rejection are antibody-mediated rejection (ABMR) and T cell-mediated rejection (TCMR). ABMR is one of the major causes of dysfunction and is characterized by the presence of donor specific antibodies (DSA) reactive to human leukocyte antigen (HLA) [1, 2, 6–8].

IFTA is an uncommon but well-known cause of graft loss. De novo glomerulonephritis has no relation to native kidney disease and commonly includes (a) membranous nephropathy (MN), (b) focal segmental glomerulosclerosis (FSGS), and (c) membrano proliferative glomerulonephritis (MPGN) [9, 10]. Calcineurin inhibitors (CNIs) used as mainstay immunosuppressive therapy can lead to renal toxicity and graft failure.

Acute tubular necrosis (ATN) is one of the leading causes of delay in recovery and delayed graft function resulting in oliguria and anuria clinically lasting more than a week after the transplant [11]. Cold ischemia time has been established as a major factor in the development of ATN [12, 13].

## 2. Materials and Methods

In this retrospective study, 163 ultrasound-guided core biopsies of live-related renal transplant cases reported, using the Banff classification (2019), from 2014 to 2019 were retrieved and reviewed. Approval from the Institutional Review Board was obtained prior to the commencement of this study. The cases with a history of live-related transplant, information regarding the original disease, latest lab results, and complete histopathological, IHC, and IF data were included in the study. Cases which had nonrelated transplant or had incomplete clinical and histopathological information were excluded.

Slides of formalin-fixed paraffin-embedded renal tissue blocks (maximum thickness 3–4 micron) were pulled out and reviewed. For light microscopy, hematoxylin and eosin along with Jones methinamine (JMS), periodic acid-schiff (PAS), and trichrome special stains were used. These three special stains (JMS, PAS, and Trichrome) of kit company Roche Ventana were performed using Ventana Benchmark Special stainer. The immunohistochemical stains (IHC) such as C4d and SV40 were performed on Leica Bond III IHC stainer with the retrieval time of 20 minutes for C4d and 40 minutes for SV40. Departmental immunofluorescence (IF) image database was consulted to review IF results of the individual cases.

Relevant clinical details of every case including history, age, sex, time since transplant, prebiopsy renal function test, urine complete examination (to look for proteinuria and hematuria), and serological parameters were obtained through the electronic medical record of the hospital information system (HIS) and telephonic calls. The mean duration of development of each pathology was estimated by calculating the time period between the transplant and biopsy.

Statistical analysis was performed using Cox regression for actuarial analysis of graft survival and logistic regression for dichotomous data, preceded by backward elimination. Data are expressed as mean ± SD unless otherwise stated.

The mean prebiopsy serum creatinine value for each etiology was calculated and categorized into three categories.

- Normal: <1.2 mg/dl
- Borderline increased: 1.2–1.5 mg/dl
- Significantly increased: >1.5 mg/dl

## 3. Results

Of the total 163 patients of live-related renal transplant, 26 (16%) were females and the rest 137 (84%) were males with the mean age of 34 ± 7 years. Most of the patients in our study were in the age group of 31–40 years (37%).

The results were studied in light of Banff classification (2019) categories. 49 of 163 cases (29.4%) were of ABMR of which 33 (20.0%) were active ABMR and 16 (9.8%) were chronic ABMR. One case (0.6%) was reported as borderline changes. 4 cases (2.5%) were diagnosed as TCMR of which only 1 (0.6%) case was of acute TCMR and 3 (1.8%) were of chronic TCMR.

IFTA was found to be the cause of transplant rejection in 15 (9.2%) cases. In the other category, recurrence of original disease was seen in 44 (27%) of the cases while the rest of 50 cases (30.7%) were of pathologies like de novo glomerulonephritis, CNI toxicity, Polyomavirus, and de novo diabetic nephropathy (Table 1).

The pathologies causing early graft rejection included active ABMR (which developed after 7.1 ± 4.7 months), acute TCMR (5.5 months), and acute tubular necrosis (after 6 ± 2.2 months of transplant). In the recurrence category, FSGS developed after 9 ± 6.1 months of transplant. Chronic AMBR was diagnosed after 17 ± 10.4 months and chronic TCMR after 22 ± 13.2 months. Late graft dysfunction was noted in CNI toxicity and IFTA (34 ± 4.7 and 35 ± 7.8months, respectively) [Table 1].

The value of serum creatinine was significantly raised in cases with evidence of rejection (ABMR and TCMR) with a mean value of 3.85 ± 0.55 mg/dl and was borderline increased in FSGS and within normal limits in the rest of etiologies like recurrence and de novo GN (Table 2).

## 4. Discussion

Renal transplant is one of the leading and time-tested treatments of end-stage renal disease throughout the world. Various etiologies like diabetic nephropathy, glomerulonephritis, interstitial nephritis, and obstructive nephropathy can lead to ESRD, and most of the time, there may be an overlap of more than one cause. Medical management and dialysis provide a temporary relief to the patient, but for long term management, the renal transplant has been adopted as a favored approach in the past few decades [1, 4]. Limited data are available on the etiologies of transplant dysfunction especially in our region, and the purpose of this original article is to contribute to the literature and help in establishing the local registry [14].

In our study, the demographic details of the recipient and donor were almost comparable to those reported from nearby countries. The donors in all of our cases were living-related compared to 97.6% living donors in an Iranian study [14] [Table 3].

**Figure 1:**
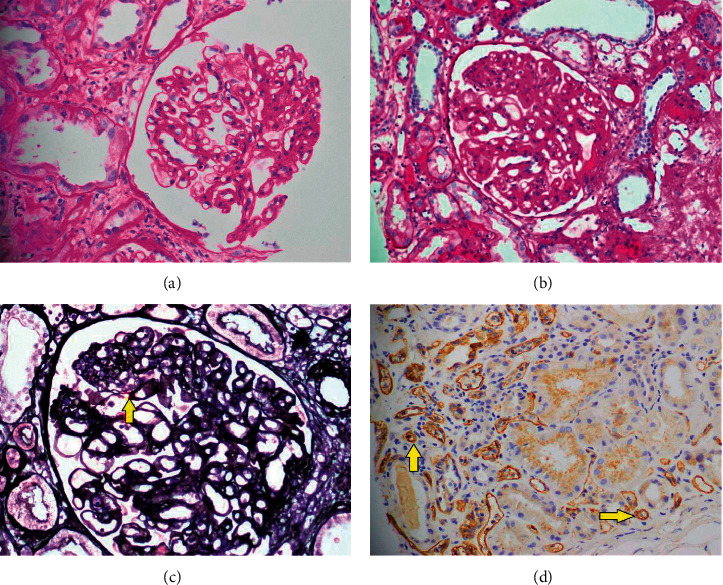
Pathological findings in chronic ABMR. (a, b) Glomeruli with patent capillary loops with endothelial cells swelling, thickened basement membranes, and increase in the mesangial matrix (periodic acid schiff (PAS) stain ×40). (c) Silver stain highlighting patent capillary loops with thickened basement membrane with duplication in ≥10% of the patent loops (arrowhead) (Jones methenamine silver (JMS) ×40). (d) Linear staining of C4d in peritubular capillaries (arrowheads) (C4d immunohistochemical stain ×40).

Acute rejection (AR) is the most fearsome outcome of any transplant [1, 2]. The relatively higher incidence of 21% found in our study as compared to 8.1% noted in earlier literature [1] is justified by the reason that all our patients have undergone transplant from a living-related donor and poor posttransplant immunosuppression is achieved due to the limited use of steroids and CNIs in our country. The incidence of ABMR has increased in the last few decades as better diagnostic techniques for the detection of antibodies have emerged [6]. Also, heavy immunization, pretransplant therapeutic strategies (blood transfusion), and retransplantation lead to sensitization and development of anti-HLA antibodies [15]. In fact, anti-HLA antibodies are already present in about 30% of pretransplant and 25% of unsensitized posttransplant patients putting them at risk of ABMR [6, 16]. Histologically, active ABMR is characterized by linear C4d staining in peritubular capillaries, microvascular inflammation, intimal or transmural arteritis, acute thrombotic microangiopathy, and fibrinoid necrosis of arteries [6, 17–19]. On histological examination, chronic ABMR has features of transplant glomerulopathy (multilayering of GBM), peritubular capillary multilayering, and arterial intimal fibrosis (Figures 1(a)–1(d)) [1, 2, 17, 20]. In the present study, 33 (20.2%) patients displayed features of active ABMR with linear C4d staining in peritubular capillaries compared to that in a study by Filippone et al. [2], in which 15% of patients developed DSA and features of active ABMR. On the other hand, 16 (9.8%) patients fulfilled the criteria of chronic ABMR in our study compared with the previous reports of the incidence rates of 1.6% to 7% [21, 22]. The mean time of development of active ABMR was 7.1 ± 4.7 months, while chronic ABMR was reported after 17 ± 10.4 months of transplant (Table 1) Only 1 (0.6%) case of borderline changes was reported.

**Figure 2:**
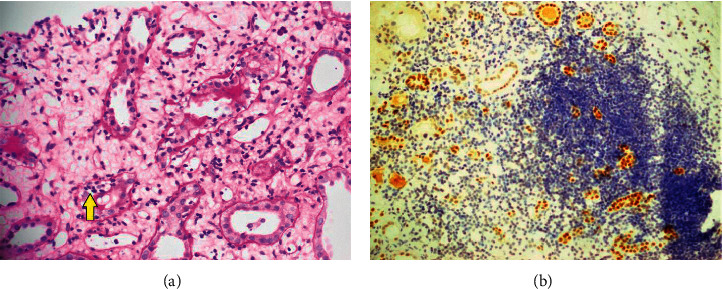
(a) Pathological findings in acute TCMR with the infiltration of inflammatory cells in the tubules and interstitium (arrowhead) (periodic acid Schiff (PAS) stain ×40). (b) SV40 immunostaining in a patient with polyomavirus nephropathy (PVN). Distal tubular epithelial cells showed diffuse nuclear SV40 positivity (SV40 immunohistochemical stain ×20).

TCMR was historically thought to be the main cause of graft dysfunction, but its incidence has now lessened due to robust induction protocols prior to transplant. Torres et al. evaluated 59 allograft biopsies and found cell-mediated rejection in 17 (29%) of cases [2, 23]. It is characterized by interstitial inflammation, tubulitis, and arteritis with various grades. There is an infiltration of *T* lymphocytes and macrophages in the tubules and interstitium (Figure 2(a)) [1, 4]. A total of 4 of 163 (2.5%) patients were found to have cell-mediated rejection which is far the less when compared to 29% in Torres et al.'s study [2, 23]. Only 1 of 163 patients was diagnosed as acute TCMR after 5.5 months of transplant. The rest of the 3 (1.8%) patients had the histopathological features of chronic TCMR (mean time of diagnosis 22 ± 13.2 months).

As per Banff classification, both the interstitial fibrosis (ci) and tubular atrophy (ct) are scored from 0 to 3 based on the percentage of respective elements in the biopsy [4]. Nankivell et al. [24] found IFTA 2/3 scores in 128 of 1138 patients (11.2%) which are almost comparable as 15 of 163 (9.2%) patients in our study suffered transplant failure solely due to IFTA.

In the “others” category of Banff classification, 27% of the graft dysfunction in our experience was from the recurrence of original pathology. In Japan, graft loss due to recurrence of disease was 1.8% [1], but recent studies point out to higher incidence of recurrence as one study reported the occurrence of recurrent GN in 24.3% of allograft patients [25]. The most common recurrent pathology was focal segmental glomerulosclerosis (FSGS) followed by membranoproliferative glomerulonephritis (MPGN) and IgA nephropathy. Recurrence rates for FSGS patients range from 30 to 60% worldwide [1]. However, in our study, only 15 (9.2%) patients suffered from FSGS. As for MPGN, 10 (6.1%) cases were reported as compared to 30–50% worldwide [1]. IgA nephropathy was also reported in the lesser percentage of cases (5.5% compared with 30–35%) when analyzed with the literature from other studies [1].

CNI toxicity is an uncommon cause of end-stage renal disease around the world (3.2 to 4.8%) [1]. In renal transplant cases, the incidence of CNI nephrotoxicity ranges from 76.4% after 1 year to 96.8% after 10 years of transplant [27]. In our study, only 15 (9.2%) patients suffered from CNI toxicity (after a mean duration of 34 ± 4.7 months posttransplant) due to limited doses of immunosuppression in the early posttransplant period owing to high treatment costs and limited affordability of the patients in our country.

We reported a single case (0.6%) of BKVN suggesting a relatively uncommon incidence in the region compared to global incidence of 5–10% of renal transplants [28]. In BKVN, there are ground glass intranuclear inclusions, nuclear enlargement, cell lysis, denudation of tubular basement membrane, IF/TA, tubulitis, and SV40 nuclear positivity (Figure 2(b)) [1, 29]. The virus is detected in about 10% of renal allografts and 2% actually develop PVN [1, 28].

It is important to distinguish between the recurrence of GN versus de novo GN as de novo GN is very uncommon and occurs in the late period (usually years) after transplant [25, 30]. A complete history regarding original disease and use of ancillary techniques, like phospholipase A2 receptor (PLA2R) antibody positivity in recurrent MN [9, 25, 31] and IgG1 deposition in de novo MN [32], is of help in this regard. Only 3 of 163 (1.9%) patients had de novo GN (2 MN and 1 FSGS) which is in accordance with the rare incidence of this etiology in graft dysfunction [9].

12 (7.4%) patients in our study were found to have ATN, and this incidence is lower than reported by Mondher et al. [12] in 2012 who found out 39 of 255 (15.29%) patients presented with ATN. This lower incidence can be attributed to live renal transplantation in our patients with lesser cold ischemia time in our general practice [11]. Arteriovenous thrombosis, which is a major differential diagnosis of ATN, is ruled out with the help of Color Doppler USG [33] and BOLD-MRI [34].

The time for the development of graft dysfunction was calculated from the date of transplant to the date of biopsy. Early graft rejection was due to active ABMR, acute TCMR, and ATN which caused graft dysfunction after 7.1 ± 4.7, 5.5, and 6 ± 2.2 months of transplant, respectively. FSGS and cortical infarction/TMA developed after 9 ± 6.1 and 8 ± 5.9 months followed by chronic ABMR and TCMR (17 and 22 months, respectively). The late causes of rejection included CNI-toxicity (34 ± 4.7 months), IFTA (35 ± 7.8 months), and de novo diabetic nephropathy (57 ± 14.5 months) (Table 1).

Prebiopsy serum creatinine levels of subjects were analyzed and divided into three major categories: normal, borderline increased, and significantly increased (Table 2).

The cases that had evidence of any form of rejection had significantly increased serum creatinine with a mean value of 3.85 mg/dl. Cases with FSGS showed a borderline increase in creatinine with a mean at 1.73 mg/dl, and the rest of etiologies like recurrence, IFTA, and de novo GN had normal mean creatinine levels (Table 2).

On follow-up, around 45% (24 of 54) patients of rejection had normalization of serum creatinine levels after appropriate treatment with intravenous immune globulin, corticosteroids, and plasmapheresis or anti-CD20 antibodies [19], while the rest (55%) required prolonged renal replacement therapy.

Overall, it was noted that most of the etiologies followed the incidence pattern of various studies around the world as mentioned in individual sections; however, there were few significant variations. Active ABMR was relatively more common in our study group (20% compared to 15%). CNI toxicity (9.2%) was less frequently noted in our study relative to global incidence (76.4 to 96.8%). ATN also had a lesser incidence in our study (7.4% compared to 15.29%).

The timing of development of various etiologies was found to be different with some causing early graft loss and others taking a longer course to develop. Also, prebiopsy serum creatinine levels can be of help in prediction of etiology of renal dysfunction even before histological diagnosis.

Limitations of our study included nonavailability of the electron microscope and relatively small sample.

## 5. Conclusion

Renal allograft dysfunction requires a correlation between clinical and histopathological findings. The timing of the development of various etiologies and the use of prebiopsy serum creatinine levels can help in the prediction of etiology of renal dysfunction even before histological diagnosis; however, renal graft biopsy remains the gold standard for a definite diagnosis. Due to the considerable diversity of allograft pathologies, a detailed understanding of Banff classification is essential.

## Figures and Tables

**Table 1:** Frequency of different etiologies (Banff category-wise) of graft dysfunction amongst the 163 cases and the mean time of development of each pathology (in months).

Banff category	Etiology	Number of cases	Mean time between transplant and diagnosis (months ± SD)
Category 1	Unremarkable	0	—

Category 2	ABMR-active	33	7.1 ± 4.7
	ABMR-chronic	16	17 ± 10.4

Category 3	Borderline TCR	1	6.8

Category 4	TCMR-Acute	1	5.5
	TCMR-Chronic	3	22 ± 13.2

Category 5	IFTA	15	35 ± 7.8

Category 6: others (recurrence)	Crescent GN	1	24
	Membranous GN	6	23 ± 8.2
	IgA	9	26 ± 7.5
	FSGS	15	9 ± 6.1
	SLE	3	21 ± 6.0
	MPGN	10	24 ± 11.7

Category 6: others	PVN	1	30
	Cortical infarction and TMA	5	8 ± 5.9
	Tubular interstitial nephritis	7	12 ± 2.3
	CNI-toxicity	15	34 ± 4.7
	De-novo diabetic nephropathy	4	57 ± 14.5
	Acute tubular necrosis	12	6 ± 2.2
	PTLD	3	24 ± 8.6
	De novo GN	3	14 ± 12.2

**Table 2:** Mean serum creatinine value of major etiologies in patients with graft impairment.

Creatinine category (serum creatinine mg/dl)	Etiology	Mean serum creatinine value ± SD (mg/dl)
Significantly increased (>1.5 mg/dl)	Rejection (all types)	3.85 ± 0.55
Borderline increase (1.2–1.5 mg/dl)	FSGS	1.73 ± 0.34
Normal (<1.2 mg/dl)	Recurrence (other than FSGS)	1.10 ± 0.20
	De novo GN	1.08 ± 0.05
	Acute tubular necrosis	1.02 ± 0.3
	CNI toxicity	0.85 ± 0.19
	**IFTA**	**0.73**

**Table 3:** Comparison of demographic features of our study with Maraghi et al.'s study.

Variable	Our study (*n* = 163)	Maraghi et al.'s Study (*n* = 461)
Age of recipient (yrs ± SD)	34 ± 7	41.80 ± 13.04
Recipient men	137 (84%)	310 (67.20)
Living donor	163 (100%)	405 (97.60)

## Data Availability

The data used to support the findings of this study are restricted by the Institutional Review Board of Shaukat Khanum Memorial Cancer Hospital in order to protect patient privacy. Data can be made available for researchers, who meet the criteria for access to confidential data, via an official request to the corresponding author (E-mail id: shaarifbashir@yahoo.com).
